# An In Vivo Whole-Transcriptomic Approach to Assess Developmental and Reproductive Impairments Caused by Flumequine in *Daphnia magna*

**DOI:** 10.3390/ijms24119396

**Published:** 2023-05-28

**Authors:** Edoardo Pietropoli, Marianna Pauletto, Roberta Tolosi, Silvia Iori, Rosa Maria Lopparelli, Ludovica Montanucci, Mery Giantin, Mauro Dacasto, Marco De Liguoro

**Affiliations:** 1 Department Comparative Biomedicine and Food Science, University of Padova, 35020 Padova, Italy; edoardo.pietropoli@phd.unipd.it (E.P.); roberta.tolosi@unipd.it (R.T.); silvia.iori@phd.unipd.it (S.I.); rosa.lopparelli@unipd.it (R.M.L.); mery.giantin@unipd.it (M.G.); mauro.dacasto@unipd.it (M.D.); marco.deliguoro@unipd.it (M.D.L.); 2 Genomic Medicine Institute, Lerner Research Institute, Cleveland Clinic, Cleveland, OH 44195, USA; montanl@ccf.org

**Keywords:** antibiotics, fluoroquinolones, flumequine, *Daphnia magna*, toxicity, phenotypical effects, gene expression, RNA-seq

## Abstract

Among veterinary antibiotics, flumequine (FLU) is still widely used in aquaculture due to its efficacy and cost-effectiveness. Although it was synthesized more than 50 years ago, a complete toxicological framework of possible side effects on non-target species is still far from being achieved. The aim of this research was to investigate the FLU molecular mechanisms in *Daphnia magna*, a planktonic crustacean recognized as a model species for ecotoxicological studies. Two different FLU concentrations (2.0 mg L^−1^ and 0.2 mg L^−1^) were assayed in general accordance with OECD Guideline 211, with some proper adaptations. Exposure to FLU (2.0 mg L^−1^) caused alteration of phenotypic traits, with a significant reduction in survival rate, body growth, and reproduction. The lower concentration (0.2 mg L^−1^) did not affect phenotypic traits but modulated gene expression, an effect which was even more evident under the higher exposure level. Indeed, in daphnids exposed to 2.0 mg L^−1^ FLU, several genes related with growth, development, structural components, and antioxidant response were significantly modulated. To the best of our knowledge, this is the first work showing the impact of FLU on the transcriptome of *D. magna*.

## 1. Introduction

In the 1970s, flumequine (FLU), obtained by inserting a fluorine atom in position C6 in the basic structure of the quinolones [1], was the first fluoroquinolone (FQ) to enter the market. This chemical modification conferred a broader spectrum of antimicrobial activity and was found to significantly increase tissue penetration. Later on, thanks to the synthesis efforts, several other FQs have been developed, some of them being even more effective than FLU, both in terms of spectrum of action and pharmacokinetic properties. Some of these FQs (clinafloxacin, gatifloxacin, grepafloxacin, norfloxacin, temafloxacin, trovafloxacin) were banned early on because of their severe adverse reactions, while others have become the drug of choice in many circumstances, even if serious side effects were occasionally reported [2]. Today, several compounds are on the market, classified into first-, second-, third-, and fourth-generation FQs [3]. They are employed in both human (ciprofloxacin, delafloxacin, levofloxacin, lomefloxacin, moxifloxacin, ofloxacin, pefloxacin, prulifloxacin, and rufloxacin) and veterinary medicine (flumequine, enrofloxacin, danofloxacin, pradofloxacin, difloxacin, marbofloxacin, orbifloxacin).

As to their mechanism of action, FQs target bacterial DNA gyrase and topoisomerase IV, which are both type II topoisomerases. They interact with the DNA-bound enzyme and lead to conformational changes that result in the inhibition of physiological enzyme activity. As a result, in the case of gyrase, the progression of the replication fork is blocked, thereby inhibiting normal DNA synthesis, whilst in the case of topoisomerase IV, the circular DNA is broken but no longer re-ligated [4]. Affinities of FQs to bacterial topoisomerases are surprisingly selective, although some sequence similarities between human and bacterial type II topoisomerases exist [5]. However, over the years, many studies have highlighted the ability of FQs to induce DNA damage even in eukaryotic organisms [6,7,8,9,10]. FLU, in particular, was found to promote liver tumorigenesis in mice, through the induction of DNA strand breaks [11]. Other authors documented its genotoxicity [12] and its contribution to the genotoxicity of contaminated waterways and groundwater [13]. Meanwhile, evidence of the side effects of FQs in humans, with serious and disabling consequences, has increased [14]; as a matter of fact, some of these effects (e.g., renal toxicity, tendinopathies) might be explained by FQ-induced epigenetic alterations in mammalian cells [15].

As FQs are broad-spectrum, widely used, and environmentally persistent antibiotics [16], their toxicity on non-target organisms is of present and future concern. Indeed, their presence in various environmental compartments has been widely reported [17]. In freshwater environments, concentrations of a few mg kg^−1^ were detected in sediments [18,19], whilst in the water column, they are generally in the range of ng L^−1^ to µg L^−1^ [20,21]. However, some exceptionally high concentrations (mg L^−1^) were reported in close proximity to drug manufacturing activities [22,23,24] and shrimp farming in Far Eastern countries [25]. FLU, in particular, has rarely been searched for, probably because its use in human medicine is rather limited; however, as a consequence of its large use in aquaculture, traces of this antibiotic (ng L^−1^) have been detected in river waters, both in France [26,27] and Italy [28]. Interestingly, a recent field study has demonstrated the persistence of FLU in marine sediments and its tendency to be taken up by benthic invertebrates [29]. Previously performed tests conducted in our laboratory on the crustacean *D. magna* evidenced that both enrofloxacin [30] and FLU [31] show a toxicity that increases over generations and is characterized by remarkable phenotypic alterations, reproduction inhibition, and mortality. Interestingly, these effects, in the case of FLU, persist after the following three generations not exposed to the drug, thereby indicating a transgenerational toxicity.

*Daphnia magna* is a model organism with a crucial advantage for genetic studies since, under controlled environmental conditions, it has a parthenogenetic reproductive cycle that allows the obtainment of genetically homogeneous clones. Considering their position in the food chain and their filter-feeding capabilities, Daphnia represent a key ecological genus that can be useful in ecotoxicity assessment and in fundamental research, as well. Furthermore, transgenerational inheritance in Daphnia is particularly relevant because the mother has been demonstrated to significantly influence the phenotypic response in the subsequent offspring. In this respect, the epigenetic patterns altered under chronic stress in these microcrustaceans can be easily monitored in the progeny and their subsequent generations [32], further increasing the utility of daphnids for ecotoxicity assessment. Epigenetic patterns in *D. magna* have been reported to be influenced by environmental stressors and passed on to subsequent generations non-directly exposed to the substance [33,34]. *Daphnia pulex* genome sequence has revealed an extraordinarily high number of genes shared with humans when compared to that of any other arthropod [35]. This genetic overlap means that, besides its relevance for ecotoxicological tests, the genus *Daphnia* could represent a useful model for predicting possible toxic effects in humans. Notably, the genome of *D. magna* has been recently assembled and characterized as never before [36]. In recent years, for all the reasons mentioned above, organisms of the genus *Daphnia* have been often employed in high-throughput omics studies to assess the molecular mechanisms of toxicity of several environmental pollutants. For instance, whole-transcriptomic studies, by means of RNA sequencing (RNA-seq), have been recently conducted in daphnids to investigate the mechanistic toxicity induced by nanoplastics [37], butyl benzyl phthalate [38], and several drugs [39,40].

The aim of the present study was to investigate the molecular mechanisms underlying FLU toxicity in *D. magna*. To reach this goal, daphnids were chronically exposed to either 0.2 mg L^−1^ (FLU-L) or 2.0 mg L^−1^ (FLU-H) of FLU. Survival, growth and reproduction endpoints were then coupled to a whole-transcriptome analysis (RNA-seq) conducted on the surviving individuals.

## 2. Results

### 2.1. Phenotypical Responses

**Figure 1:**
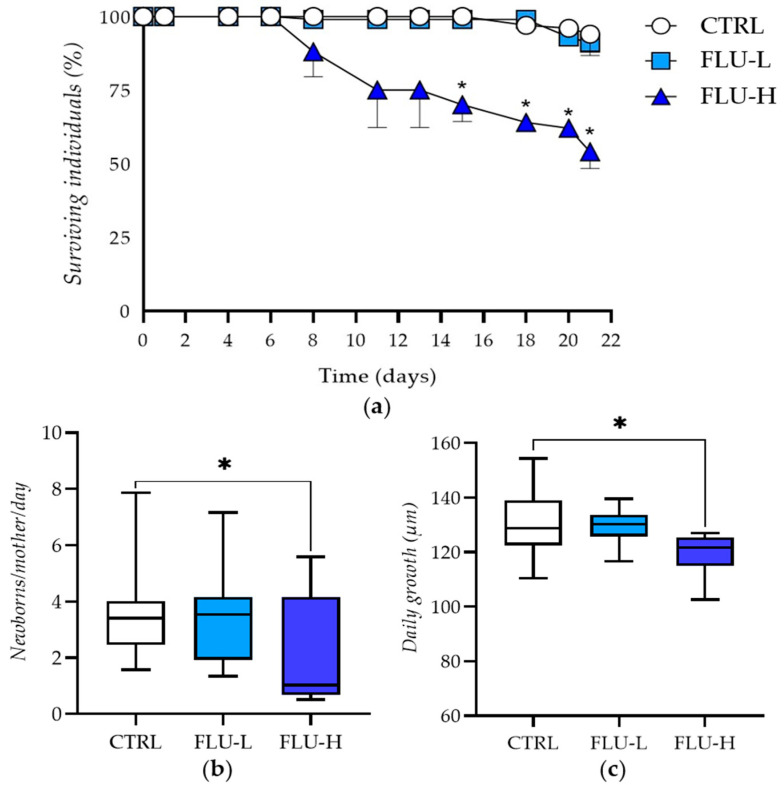
Effects of flumequine on *D. magna* F0 generation following a chronic exposure (21 days). In the three experimental groups, the endpoints considered were survival rate (**a**), reproduction rate expressed as daily new births per mother organism at the start of the test (**b**), and growth rate expressed as daily growth during the 21-day test period (**c**). Data are given as mean ± standard error (SEM) of two independent batches of daphnia, each consisting of 50 organisms. *: *p* ≤ 0.05 (one-way ANOVA followed by Tukey’s test). FLU-L: 0.2 mg L^−1^; FLU-H: 2.0 mg L^−1^.

Statistically significant differences in phenotypic traits were found when comparing FLU-H vs. CTRL (Table 1, Figure 1).

The highest FLU concentration, compared to CTRL, significantly increased the mortality rate by 40% (Table 1, Figure 1a), decreased the reproductive capacity by 46.5% (Table 1, Figure 1b), and reduced the daily growth by 8.82% (Table 1, Figure 1c). As a general consideration, it should be noted that growth inhibition is underestimated because measurements were made only in surviving individuals, without considering organisms that died during the test, which were remarkably underdeveloped.

Besides assessing mortality, reproduction, and growth, animals were monitored daily. Lethargic behavior was observed in many daphnids of the FLU-H group, resulting in reduced feed consumption and consequent accumulation of algae on the bottom of the vessel. However, ephippia (winter eggs) never appeared during the 21-day experiment, thereby indicating that the parthenogenetic cycle was steadily maintained.

### 2.2. Transcriptomic Effects of FLU in D. magna

#### 2.2.1. Differentially Expressed Genes (DEGs)

For each experimental group (CTRL, FLU-L, and FLU-H), three pools of *D. magna* were considered, each consisting of six organisms. Therefore, there were three biological replicates per group, but the overall biological variability represented in this study consisted of eighteen daphnids per experimental condition. A total of nine tagged RNA-seq libraries were sequenced. From each sequenced pool, ~30 million reads (on average) passed the quality control and trimming steps. An average of 88.74% reads uniquely mapped against the *D. magna* reference genome, while an average of 10.58% mapped against multiple loci. Overall, a mapping percentage of 99.32% was achieved (Table S1). Notably, we observed that the reference genome, consisting of 24752 genes coding for 19748 proteins, is still poorly annotated, as shown by the high percentage (i.e., 58%) of uncharacterized proteins.

**Figure 2:**
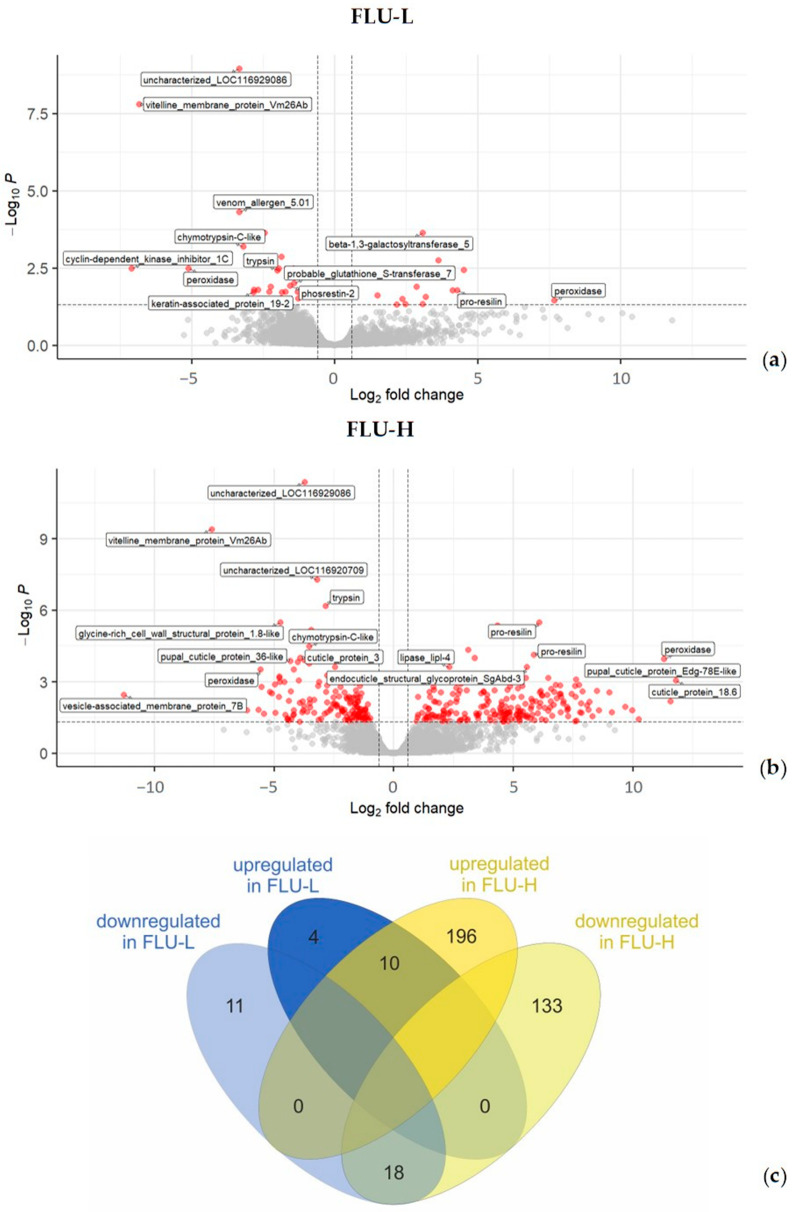
Differentially expressed genes. The figure reports volcano plots of the FLU-L (**a**) and FLU-H groups (**b**), and a Venn diagram summarizing DEGs (**c**). In (**a**,**b**): logarithms (base 2) of fold changes are reported on the x axis; logarithms (base 10) of the corrected *p*-values (FDR) are reported in the y axis; dashed lines set the threshold of significance, FDR ≤ 0.05, and log fold-change, LFC ≥ 0.6 or ≤ −0.6; significantly regulated genes are shown as red dots in the upper left and upper right quadrants (down- and upregulated genes, respectively); non-significantly regulated genes are represented as grey dots. In (**c**): the Venn diagram shows the number of differentially expressed genes in each group, highlighting the extent of genes significantly up- or downregulated by both FLU concentrations. FLU-L: 0.2 mg L^−1^; FLU-H: 2.0 mg L^−1^, CPM: counts per million.

As shown in Table 1, FLU caused maximal transcriptional changes at the highest concentration (FLU-H). Specifically, as reported in Figure 2a and Table 1, FLU-L and FLU-H modulated a total of 43 and 357 DEGs, respectively. Out of the 43 DEGs affected by the lowest FLU concentration, 14 were upregulated and 29 downregulated. As to FLU-H, 206 DEGs were upregulated and 151 downregulated (Figure 2b, Table 1). Interestingly, two-thirds of the FLU-L DEGs were shared with FLU-H (Figure 2c, Figure S1). The output of the differential expression analysis is detailed in Table S2.

#### 2.2.2. Functional Enrichment Analysis

The DEGs were subjected to a functional enrichment analysis to reveal whether exposure to FLU can cause changes to specific pathways (Table S3). The Gene Ontology (GO) enrichment analysis showed 23 enriched GOs for the FLU-H group, whereas, given the low number of DEGs, only one GO was enriched in the FLU-L group, namely the “Egg chorion” (GO:0042600, 2 genes).

**Figure 3:**
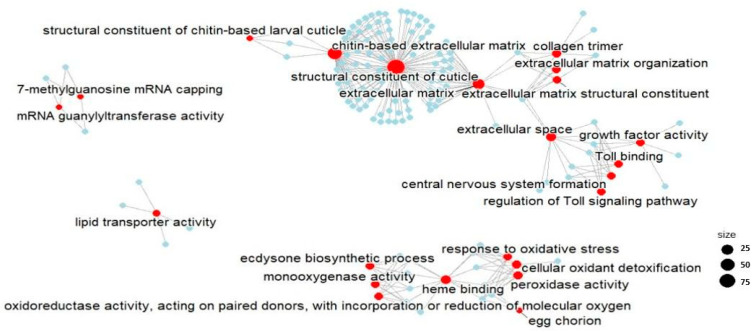
Gene Ontology enrichment analysis. The gene concept network reports the enriched pathways in the list of differentially expressed genes resulting from the comparison FLU-H vs. CTRL. Individual genes are represented in blue dots, while GOs are represented in red dots. The size of the GO points indicates the number of significantly enriched genes belonging to the specific GO.

As concerns FLU-H significant results, the complex association between the enriched pathways was extracted preparing a gene concept network that depicts the linkages of genes and GO terms and shows them as a network (Figure 3). The network showed that most of the DEGs are related to GOs involved in developmental and growth processes. Indeed, the most enriched GO terms were “structural constituent of cuticle” (GO:0042302, 97 genes), “chitin-based extracellular matrix” (GO:0062129, 50 genes), “extracellular matrix” (GO:0031012, 28 genes), “extracellular space” (GO:0005615, 10 genes), “collagen trimer” (GO:0005581, 7 genes), “Toll binding” (GO:0005121, 6 genes), “central nervous system formation” (GO:0021556, 6 genes), “growth factor activity” (GO:0008083, 6 genes), “regulation of the Toll signalling pathway” (GO: 0008592, 5 genes), “extracellular matrix organization” (GO:0030198, 5 genes), “structural constituent of the extracellular matrix” (GO:0005201, 5 genes), and “structural constituent of the larval chitin-based cuticle” (GO:0008010, 3 genes).

In addition, it was observed that FLU exposure affected some GO terms involved in the response to external stressors, such as “heme binding” (GO:0020037, 12 genes), “peroxidase activity” (GO:0004601, 7 genes), “cellular oxidant detoxification” (GO:0098869, 7 genes), “response to oxidative stress” (GO: 0006979, 6 genes), “oxidoreductase activity acting on paired donors with incorporation or reduction in molecular oxygen” (GO:0016705, 6 genes), “monooxygenase activity” (GO:0004497, 6 genes), “ecdysone bio-synthetic process” (GO:0006697, 5 genes), and “egg chorion” (GO:0042600, 3 genes).

Moreover, FLU was shown to affect some additional pathways: those of the transcriptional machinery, such as the GO terms “mRNA guanylyltransferase activity” (GO:0004484, 3 genes) and “7-methylguanosine mRNA capping” (GO:0006370, 3 genes), and of lipids transport as well, such as lipid transporter activity (GO:0005319, 4 genes).

#### 2.2.3. Gene Set Enrichment Analysis (GSEA)

GSEA is an excellent tool for confirming what was observed in the GO enrichment analysis. At the same time, this analysis allows the identification of potential effects on other gene sets, since it considers the whole transcriptional profiles, also comprising the non-statistically significant mRNA changes.

**Figure 4:**
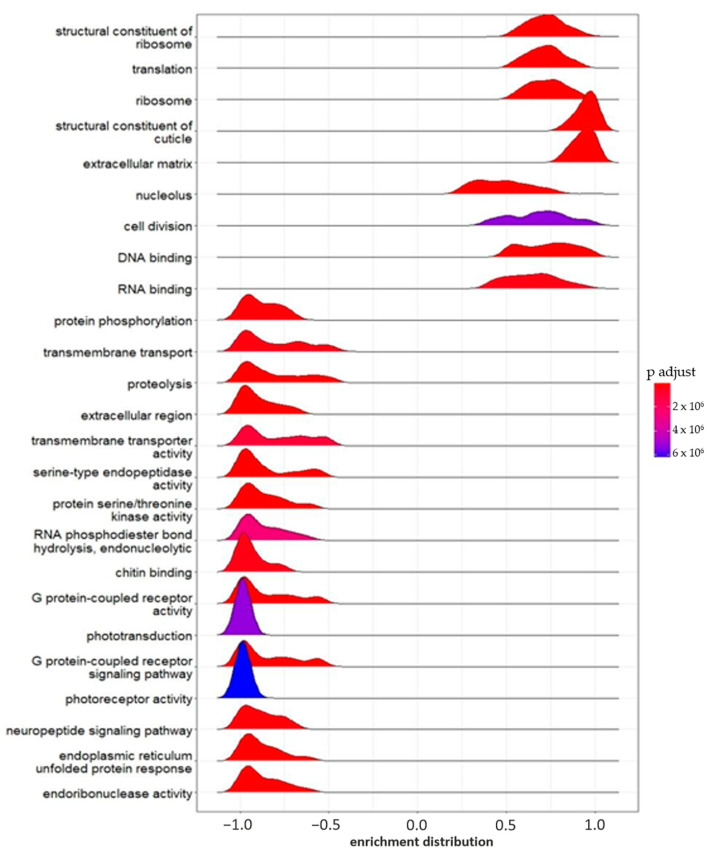
GSEA enrichment (flumequine 0.2 mg L^−1^). Ridge plot of the 25 most significant pathways enriched in the FLU-L group by Gene Set Enrichment Analysis (GSEA). The color gradient represents the adjusted significance level (p.adjust), according to the Benjamini–Hochberg method.

The GSEA showed a total of 179 enriched gene sets in the group exposed to FLU-L (Table S4). As shown in the ridge plot in Figure 4, several of these gene sets had also been found in the GO enrichment analysis of DEGs found in the FLU-H vs. CTRL comparison. Most likely, although below the threshold of significance, some genes belonging to those gene sets were also modulated by the lowest FLU concentration.

Among the enriched gene sets, we highlight the ones involved in structural composition and development, such as the structural constituent of the cuticle (GO:0042302, 253 genes), the extracellular region (GO: 0005576, 259 genes), chitin-based extracellular matrix (GO:0062129, 110 genes), extracellular matrix (GO:0031012, 98 genes), chitin binding (GO:0008061, 95 genes), and cell division (GO:0051301, 51 genes).

In agreement with the GO enrichment analysis of DEGs of the FLU-H group, some gene sets involved in the stress response were enriched; for example, the “intrinsic apoptotic signaling pathway in response to endoplasmic reticulum stress” (GO: 0070059, 25 genes), the “ecdysone biosynthetic process” (GO:0006697, 20 genes), “monooxygenase activity” (GO:0004497, 58 genes), and “intramolecular oxidoreductase activity that transposes S-S bonds” (GO:0016864, 6 genes).

Several gene sets involved in light perception were inhibited, such as “photoreceptor activity” (GO:0009881, 24 genes), “phototransduction” (GO:0007602, 27 genes), “visible light sensing” (GO:0009584, 19 genes), “visual perception” (GO:0007601, 30 genes), and “cellular response to light stimulus” (GO:0071482, 19 genes) (Figure 4).

**Figure 5:**
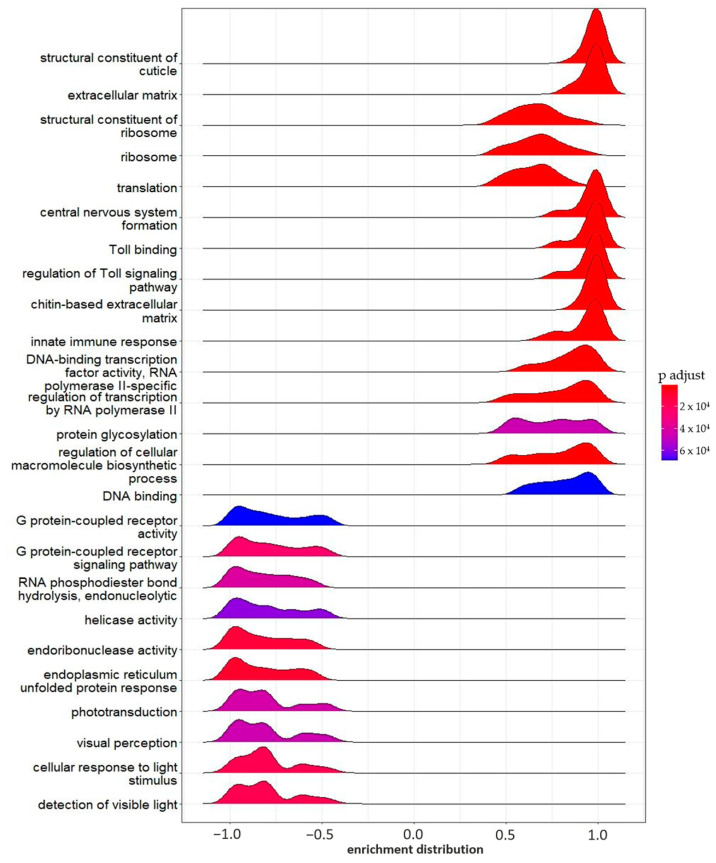
GSEA enrichment (flumequine 2 mg L^−1^). Ridge plot of the 25 most significant pathways enriched in the FLU-H group by Gene Set Enrichment Analysis (GSEA). The color gradient represents the adjusted significance level (p.adjust), according to the Benjamini–Hochberg method.

The GSEA carried out on FLU-H vs. CTRL transcriptional profiles reported 64 enriched gene sets (Figure 5). Several gene sets related to development and growth were highly enhanced; some examples are “structural constituent of cuticle” (GO:0042302, 253 genes), “extracellular matrix” (GO:0031012, 97 genes), “central nervous system formation” (GO: 0021556, 30 genes), “regulation of Toll signalling pathway” (GO:0008592, 30 genes), “chitin-based extracellular matrix” (GO:0062129, 110), and “growth factor activity” (GO:0008083, 49 genes). Furthermore, an activation was also observed of gene sets related to the stress response, among which the most significant one is “innate immune response” (GO:0045087, 31 genes).

Among the 25 most significant gene sets, likewise to the FLU-L group, an inhibition is observed with regard to light perception. These gene sets include “detection of visible light” (GO:0009584, 19 genes), “cellular response to light stimulus” (GO:0071482, 19 genes), “visual perception” (GO:0007601, 30 genes), “phototransduction” (GO:0007602, 27 genes), “protein-chromophore linkage” (GO:0018298, 30 genes), and “photoreceptor activity” (GO:0009881, 24 genes). As observed in GO analyses, factors related to gene transcription machinery are enriched, and in particular, “regulation of transcription by RNA polymerase II” (GO:0006357, 238 genes), “DNA-binding transcription factor activity, RNA polymerase II-specific” (GO:0000981, 138 genes), “mRNA guanylyltransferase activity” (GO:0004484, 5 genes), and “7-methylguanosine mRNA capping” (GO:0006370, 8 genes) are inhibited gene sets.

**Figure 6:**
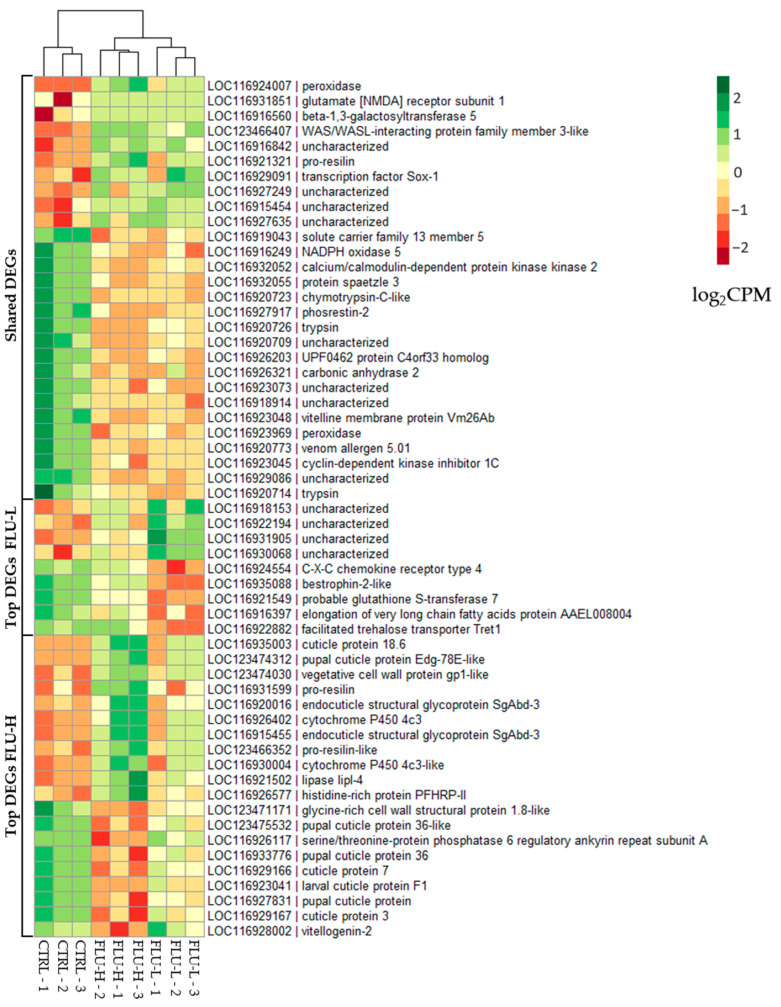
Normalized expression levels of top and shared Differentially Expressed Genes (DEGs). Heatmap of the 30 most significant DEGs in the comparisons FLU-L vs. CTRL and FLU-H vs. CTRL. Shared DEGs resulting from the two comparisons have also been reported. Top DEGs that were also listed among the shared DEGs have been reported only once, specifically among the shared DEGs. Data are expressed as normalized log2CPM (count per million).

Focusing on the DEGs of greatest interest resulted in each pairwise comparison; Figure 6 summarizes the gene expression level of the 30 most significant DEGs in each experimental group and that of the shared DEGs.

As reported earlier, the FLU-L group has only a few significant DEGs (i.e., 43), and 15 of them have unknown biological function (i.e., they are annotated as “uncharacterized locus”). It is worth noting that, 8 out of these 15 genes are included in the list of 30 top DEGs (Figure 6); thus, unfortunately, we were not allowed to infer their putative role in response to FQ. These include the uncharacterized LOC116927249 (LFC = 4. 52), LOC116918153 (LFC = 4.14), LOC116922194 (LFC = 3.19), LOC116930068 (LFC = 3.09), LOC116916842 (LFC = 2.88), LOC116915454 (LFC = 2.37), LOC116927635 (LFC = 2.18), and LOC116931905 (LFC = 1.51). Among the genes with a putative function, a clear and unambiguous response was not observed. Within the list of upregulated genes with a known biological function, we found peroxidase (LFC = 7.69), transcription factor Sox-1 (LFC = 4.73), pro-resilin (LFC = 4. 30), WAS/WASL-interacting protein family member 3-like (LFC = 3.63), beta-1,3-galactosyltransferase 5 (LFC = 3.08), and glutamate receptor subunit 1 [NMDA] (LFC = 2.48). Among the downregulated genes, we found facilitated trehalose transporter Tret1 (LFC = −1.28), a probable glutathione S-transferase 7 (LFC = −1.41), UPF0462 C4orf33 homolog protein (LFC = −1.42), phosphrestin-2 (LFC = −1.55), bestrophin-2 (LFC = −1.55) 55), bestrophin-2-like (LFC = −1.71), spaetzle protein 3 (LFC = −1.84), RNA polymerase II DNA-directed subunit RPB7 (LFC = −1.88), calcium/calmodulin-dependent protein kinase kinase 2 (LFC = −1.95), trypsin (LFC = −1. 99), trypsin (LFC = −2.23), solute carrier family 13 member 5 (LFC = −2.29), C-X-C chemokine receptor type 4 (LFC = −2.67), keratin-associated protein 19-2 (LFC = −2.84), kymotrypsin-C-like (LFC = −3.19), venom allergen 5. 01 (LFC = −3.33), very long-chain fatty acid elongation protein AAEL008004 (LFC = −3.58), peroxidase (LFC = −5.11), vitellin membrane protein Vm26Ab (LFC = −6.83), and cyclin-dependent kinase inhibitor 1C (LFC = −7.09) (Figure 6).

Consistent with the functional analyses, among the top DEGs of the FL-H group, we observed that there were several genes involved in the structural formation of the organism regardless of the sense of expression, such as several cuticle proteins, e.g., cuticle protein 18.6 (LFC = 11.81), pupal cuticle protein Edg-78E-like (LFC = 8.78), larval cuticle protein F1, 3, and 7 (LFCs = −2.43, −3.88, −3. 99), pupal cuticle proteins 36 and 36-like (LFC = −4.55, −4.30), and other proteins that give stability or flexibility to the cuticle such as two endocuticle structural glycoproteins SgAbd-3 (LFCs = 7. 76, 6.99) and two pro-resilins (LFCs = 7.91, 6.48). In addition, we observed a tendency of overexpression of genes involved in the stress response and detoxification such as a peroxidase (LFC = 11.32), cytochrome P450 4c3-like (LFC = 6.97), cytochrome P450 4c3 (LFC = 6.79), histidine-rich protein PFHRP-II (LFC = 4.35), and peroxidase (2) (LFC = −5–56) (Figure 6).

Additionally, and in agreement with phenotype results, a downregulation of two key genes involved in reproduction was observed, i.e., vitellogenin-2 (LFC = −1.85) and vitelline membrane protein Vm26Ab (LFC = −7.59).

Regarding the shared DEGs, we detected genes potentially involved in the stress response, for which a dose-dependent increase in fold-change was observed; among these, two peroxidases are noteworthy. The same trend (i.e., the higher the concentration, the greater the fold-change) was shown by some structural genes of *D. magna*, such as pro-resilin (LFC = 4.30 and LFC= 5.87), and for the vitelline membrane protein Vm26Ab, a key element in the composition of eggs, whose LFC increases from −6.83 (FLU-L) to −7.59 (FLU-H) (Figure 6).

#### 2.2.4. Targeted Gene Expression

To validate RNA-seq data, we measured the mRNA levels of five DEGs (i.e., cuticle protein 18.6, larval cuticle protein 2-like, larval cuticle protein F1, vitelline membrane protein Vm26Ab, vitellogenin 2) using a targeted confirmatory quantitative real-time PCR (qPCR) approach. As shown in Figure S2, the obtained qPCR data corroborated the RNA-seq data, since the two technical approaches showed the same trend of expression. As to cuticle protein 18.6 and larval cuticle protein 2-like, qPCR results showed a conserved net upregulation even if the high standard deviation between qPCR biological replicates prevented results from being statistically significant.

## 3. Discussion

Antibiotics play a key role in today’s society both from a public health and economic point of view. FLU is among the authorized antibiotics in veterinary medicine and it is widely used, especially in aquaculture. This first-generation fluoroquinolone antibiotic is effective as it is able to stop bacterial proliferation by impairing the replication of their genetic material. Given the scarcity of data on the potential toxicity of FLU in the aquatic environment, the present study focused on confirming FLU toxicity on *D. magna*, already observed by De Liguoro and colleagues [31], by expanding the toxicological framework through transcriptomic investigations. As a starting point, we used the same concentration (2.0 mg L^−1^) used in that study, but we also assayed another concentration of a lower order of magnitude (0.2 mg L^−1^).

In the previous study mentioned above, run in our laboratory, it was observed that *D. magna* was not significantly affected by FLU 2.0 mg L^−1^ when considering lethality (20%), reproduction inhibition (20.6%), and daily growth inhibition (−1.2%) [31]. Conversely, in the current study, the mortality rate observed in the group exposed to FLU 2.0 mg L^−1^ was 40% higher than in CTRL. Furthermore, the reproduction and growth rates were significantly inhibited by 46% and 9%, respectively. The differences observed between the two studies are most likely explained by the different design of the experiments (50 + 50 daphnids in two vessels in the current, versus 10 daphnids kept individually in the previous one). Moreover, the clones of *D. magna* used in the two experiments were different; indeed, it has previously been shown that different clones of *D. magna* can show different degrees of sensitivity when exposed to xenobiotics [39,40,41,42,43,44,45].

*D. magna* has been largely used as a model species to study the whole transcriptional changes triggered by diverse classes of pharmaceuticals, such as anticancer, neuroactive and acaricide drugs providing important insights into the mode of action of these compounds [46,47,48]. In the present study, we showed that the chronic exposure to an antibiotic (FLU, 2.0 mg L^−1^) alters the expression of several genes involved in daphnids structural, developmental and growth pathways, and particularly of genes encoding several chitin-based cuticle proteins, which are fundamental to most integuments. As a confirmation of this molecular evidence, organisms exposed to FLU-H were smaller, and in most cases these organisms died during the exposure. Interestingly, similar phenotypic and transcriptomic changes were observed in *Daphnia pulex* exposed to polystyrene nanoplastics [49]. Although the specific function of each arthropod cuticle protein is still unknown, they are reported to play an essential role, as their function and composition can be altered depending on their location within the body and the role played by the tissue [50]. The exoskeleton is a component of the body of arthropods that requires great mechanical strength and rigidity.

To achieve such rigidity, the chitin of the outer cuticle is enriched with reinforcing proteins among which we can find keratin, sclerotized proteins, or calcite. Interestingly, one of the biochemical processes of exoskeleton reinforcement is sclerotization, which consists of the enzymatic introduction of quinones into the cuticle composition [50]. Due to the lack of knowledge of patterns and gene interactions in arthropods, it is not possible to know precisely and with certainty what is occurring at the biochemical level in the outermost layer of the cuticle. However, it might be possible that fluoroquinolones, such as FLU or its metabolites, may accidentally interfere with the sclerotization process as quinone surrogates.

Our transcriptomic data also revealed an important downregulation of keratin gene in FLU-H daphnids, which might result in thickening deficiency of the exoskeleton and a lower protection towards external insults. In *D. magna* exposed to FLU, the lack of hardening proteins might have been compensated by the substantial increase observed in the mRNA levels of genes coding for elasticizing proteins such as pro-resilin, a protein that is essential in making the cuticle elastic [51], and collagens. Collagens are associated with the reinforcement and support of the cuticle; it has been previously demonstrated that genetic mutation of genes encoding collagen in *C. elegans* might cause exoskeletal defects that may result in shape alterations of the animal [52]. This molecular evidence is supported by our in vivo observations. As a matter of fact, FLU-exposed daphnids had a soft and discolored body, not maintaining the normal shape.

Linked to resilin, there is the endocuticle protein, representing the innermost layer of the procuticle; this thin, flexible layer of chitin is anchored to the basement membrane, i.e., the deepest layer, or rather the non-cellular internal membrane of the integument through adhesive proteins. The endocuticle plays a key role in the molt as it tends to harden before molting to facilitate procuticle separation and is the first chitin layer formed after molting. In this regard, we observed a great upregulation of genes involved in endocuticle protein biosynthesis, i.e., endocuticle structural glycoproteins: ABD-4, SgAbd-1, SgAbd-2, SgAbd-3, SgAbd-4. Accordingly, the GSEA pointed out the significant activation of the GO terms “structural constituent of cuticle” and “chitin-based extracellular matrix”. Endocuticle proteins are known to herald a molting phase in Daphnia. Likewise, Ecdysone-dependent gene 78E (Edg78E), upregulated in the present study, is generally involved in molting, being highly expressed in the pre-molt pupal stages [53]. It has been reported that molting is the key factor controlling the growth of daphnids [54]. In the present study, mRNA changes induced by FLU seem to support molting, but in vivo we did observe a decrease in growth rates. This seeming contradiction might be explained by the fact that even if these genes, and probably the relative proteins, were induced, they were not sufficient to support Daphnia’s complete life cycle and growth. Indeed, for Daphnia growth, sclerifying proteins such as keratins and adult-specific rigid cuticular proteins are also fundamental, because they serve in cuticle thickening after molting. Notably, in the present study, most of the genes coding for sclerifying proteins were downregulated by FLU.

Peroxidases are generally known as scavengers of reactive oxygen species (ROS), and consequently they are implicated in the antioxidant response [55,56]. Importantly, it is worth mentioning that arthropods can use peroxidase as a sclerifying agent [50]. In our study, we observed a modulation in the expression of five peroxidases whose function, considering the limited biomolecular knowledge of arthropods, is still unknown. Nevertheless, a significant increase in the mRNA expression was observed in four out of five peroxidases, suggesting their possible involvement in the antioxidant response, as already observed by Zhang and colleagues following exposure of *D. pulex* to polystyrene nanoplastic [49]. Conversely, only one peroxidase was significantly inhibited. It could be hypothesized that this isoform, having a trend in agreement with other sclerifying proteins, is involved in cuticle thickening.

As stated above, although molting genes (e.g., endocuticle structural glycoproteins, Edg78E) were induced by FLU, an increased growth rate was actually not observed in vivo. Intriguingly, our molecular results also demonstrated an overall downregulation of sclerifying genes. This might result in incapacity of cuticle hardening in Daphnia, thus hampering the post-molting phase. It might be then speculated that the decrease in growth rate is due to the blockage of the Daphnia life cycle.

Interestingly, the gene encoding for fatty acyl-CoA reductase was among the genes showing the highest fold-change increase. This reductase seems to be crucial for the synthesis of several insect cuticular hydrocarbons (CHCs), which in turn are involved in protecting the insect from desiccation [57]. The study by Finet and colleagues also emphasized the rapidity in the modulation of fatty acyl-CoA reductase expression during the adaptive response to different environmental conditions. Fatty acyl-CoA reductase was also reported to play a crucial role in the generation of CHCs and waxy filaments in the cotton mealybug [58]. In the same study, it has been postulated that the gene coding for this reductase might contribute to the protective functions of the waxy layer, allowing water retention and representing a barrier against external agents, including chemicals. To prove the centrality of this gene, Tong and co-authors [58] reduced the expression of mealybug fatty acyl-CoA reductase by RNA interference, resulting in a reduction in CHC contents in the waxy layer and an increased mortality by desiccation.

Among the genes related to growth and development, CEBPA, which has been reported to have an important role in body weight homeostasis in humans [59], was shown to be affected by the treatment. Specifically, it was downregulated by the highest dose of FLU, in accordance with the impaired growth and body size observed at the phenotypic level.

Overall, our molecular results support the hypothesis that FLU might deeply interfere with the regulation of molting, growth, and development in *D. magna*.

The cytochrome P450 (CYP) superfamily of drug metabolizing enzymes in insects as well as in all the kingdoms of life are the hinge of important oxidative reactions of both xenobiotics and endogenous compounds. This broad class of enzymes includes over 360 families and more than 3100 enzymes [60,61,62]. Among them, in this study we observed the downregulation of CYP3A41. This enzyme has already shown a similar trend in mice liver as a consequence of toxicant exposure (i.e., 1,4 bis 2-(3,5-dichloropyridyloxybenzene)) [62]. In addition, the gene coding CYP4C3 was highly upregulated as a consequence of FLU exposure. Among the various CYP families, this gene has been classified into the oxidoreductase activity sub-category. A similar trend of expression has already been observed in the prawn *Marobrachium nipponense* as a consequence of sulfide toxicity [63] and in *D. magna* after exposure to ethylene-acrylic acid copolymer [64]. However, due to the limited amount of studies on gene expression after FQ exposure, and given the difficulty of identifying and determining the function of each CYP in non-human model species [65], it is not possible to suggest specific roles of these CYPs in *D. magna* exposed to FLU.

The glutathione system GSSG/2GSH can be considered the cell’s primary redox buffer [66]. The NADPH/NADP^+^ system, in turn, is considered the primary source of reducing equivalents for the glutathione system. Therefore, NADPH has been considered an indirect co-antioxidant, as it acts as a reducer of the oxidized form of the antioxidant [67]. In conditions of cellular homeostasis, NADPH oxidase (NOX) tends to catalyze the transfer of electrons from NADPH to the oxygen molecule (O_2_) to generate the superoxide radical H_2_O_2_ [68]. In our study, we observed a downregulation of NOX 5, which might be related to an already elevated level of reactive oxygen species (ROS) in cellular compartments, due to FLU exposure.

Besides the effect of FLU on daphnids’ structural, developmental, and antioxidant genes, a further concomitant factor that could explain the decreased growth rate observed in the FLU-H group is the difficulty in digesting food and transporting energy within the body. Indeed, the transcriptomic analysis revealed the modulation of genes involved in digestion and energy transport, such as trypsin, which was mainly downregulated. Trypsin is a protease whose function is to hydrolyze large polypeptides to form lysine and arginine, essential amino acids for the body [69]. Another group of proteins involved in the transport of essential substrates, whose expression was influenced by FLU exposure, is the ATP-binding cassette (ABC) transporters, specifically the G20 ABC. ABC transporters are a superfamily of membrane proteins with several functions, among which is the role of converting energy gained from ATP hydrolysis into trans-bilayer movement of indispensable cellular substrates either into or out of the cytoplasm [70].

In addition, we observed the differential expression of several genes involved in neuroendocrine signaling; among those being downregulated, there was the calcium-activated chloride channel regulator, crucial for the depolarization of neurons [71], and glutamic acid-rich proteins and glutamate receptor subunit 1 (NMDA), the main excitatory neurotransmitter of the central nervous system and their specific receptors [72]. Regarding upregulated genes, we found neuropeptide 31, which regulates many physiological activities in flies [73], neurotrophin 1, whose upregulation was previously observed in mice after the exposure to toluene [74], SRY-Box Transcription Factor 1 (SOX1), crucial for nervous system development in arthropods [75], and phosrestin 2, which is involved in photoreceptor function [76].

It is also essential to discuss the effects of FLU on reproduction. Phenotypically, a 46% reduction in reproduction rate was observed in the FLU-H group, whilst no effect was observed at the lower concentration. In agreement with the phenotypic analysis, the expression levels of genes related to reproduction showed no difference in the FLU-L group compared to the control group, except for vitelline, which was highly downregulated; conversely, in FLU-H, several genes involved in the reproduction pathway were altered in their expression profile. Among the most relevant molecular effects on reproduction, a significant downregulation of vitellin (Vn) and vitellogenin 2 (Vtg2) was observed. In crustacean species, reproduction is based on oogenesis. In this process, the oocytes are enriched with yolk protein, leading to an increase in diameter. The standard form of yolk stored in oocytes is Vn which represents a source of nutrition for developing embryos. Vn is synthesized intraovarian from vitellogenin (Vtg), enriched with polysaccharides and lipids [77]. Conversely, Vtg is synthesized in the hepatopancreas and transported to the oocytes via the hemolymphatic system [78]. These two proteins are therefore interrelated and are both essential for reproductive activity. Interestingly, Vn is known to be a key protein for egg fertility [79] and its downregulation is in agreement with the inhibition of reproduction reported in vivo. Hannas and colleagues [80] reported that during the period of ovarian oocyte maturation of *D. magna,* Vtg2 mRNA levels increase, while they tend to decrease between 24 and 48 h post-molt, in preparation for the next exuviation. This Vgt2 level drop is always correlated to the endogenous re-increase in ecdysteroidal hormone. Interestingly, they also reported that among several chemicals studied, some of them, such as cyproterone acetate, acetone, triclosan, and atrazine, showed ecdysteroidal activity, suppressing Vgt2 levels [80].

Although FQs are antibiotics largely used both in human and veterinary medicine, little is known about their possible toxic effects on non-target species. Research is only recently being developed in this respect. In 2009, Fagutao and co-workers tested the toxicity of the first-generation fluoroquinolones oxolinic acid in black tiger shrimp, *Penaeus monodon,* revealing the downregulation of immune-related gene expression [81]. Du and coworkers showed that American shad exposed to enrofloxacin (ENR), another fluoroquinolone widely used in veterinary medicine, increases body weight through dysregulation of gut metabolism (963 DEGs) and induction of lipid accumulation [82]. Exposure of Zebrafish larvae to a mixture of sulfamonomethoxine (SMM), cefotaxime sodium (CFT), tetracycline (TC), and ENR resulted in a reduction in body length and an increase in reactive oxygen species (ROS) content. KEGG analysis revealed several DEGs involved in steroid biosynthesis [83]. Luan and colleagues investigated the effects of ENR on the rat liver, finding significant damage to the hepatocytes, and following whole-transcriptome analysis, 208 DEGs were revealed, a large proportion of which were involved in cytochrome biosynthesis [84]. The toxicity of the mixture of ibuprofen, ciprofloxacin, and flumequine has been recently evaluated on the crayfish *Procambarus clarkii* [85]. The authors stated that exposure to the antibiotic mixture can affect several cell functions, such as biotransformation and detoxification processes, by modifying the expression of genes encoding antioxidant enzymes [85]. In *P. clarkii* exposed to gatifloxacin sulphide, a fourth-generation fluoroquinolone, apoptosis of hepatopancreatocytes was accelerated. Authors also reported that gatifloxacin was responsible for 84 DEGs, annotated to 178 known KEGG pathways, of which the most important were steroid hormone biosynthesis and chlorophyll metabolism [86]. More recently, reduction in spematogonial weight, spermatogonial tissue damage, and decrease in testosterone (T) gene expression have been shown in zebrafish, suggesting that ciprofloxacin (CIP) could affect endocrine signaling pathways [87].

In this study, the effects of FLU exposure were evaluated in *D. magna*. Our results showed that direct exposure to the FLU higher dose causes impairments in survival, growth, and reproduction, both at the phenotypic and molecular levels. In addition, although the FLU lower dose showed no statistical phenotypic differences, transcriptional modulation of genes involved in antioxidant response, growth, and reproduction were indeed observed.

Finally, it should be emphasized that, despite the growing interest in sequencing the DNA of most eukaryotic species (e.g., Earth BioGenome Project), some important non-mammalian species still have a poorly annotated genome. This is the case of *D. magna*. Therefore, given its prominent role as a model species in aquatic toxicology and human research, further efforts for a more accurate genome annotation are needed.

## 4. Materials and Methods

### 4.1. Chemicals

Analytical-grade flumequine (FLU), CAS number: 42835-25-6 and purity ≥98%, was supplied by Sigma-Aldrich (Milan, Italy). A 100 mg L^−1^ stock solution in Rocchetta© still mineral water (pH 7.6, dry residue 181.6 mg L^−1^) was prepared by gently stirring overnight at 37 °C. The pH was measured with a BASIC20 pH meter (CRISON, Carpi, Italy) and then the solution was stored in the dark at 4 °C until the beginning of the test. The stability of FLU in fresh and salt water has been repeatedly demonstrated [29,88,89,90]. As previously shown in our laboratory [31], under the conditions of the present experiment (48 h renewal, temperature 20 ± 1 °C, 100 lux), the degradation of FLU is negligible. Therefore, test results were based on the nominal concentration (2.0 mg L^−1^ and 0.2 mg L^−1^).

### 4.2. Test Organism and Culture Conditions

*D. magna* were purchased from a local breeder. Clones were subjected to DNA barcoding, confirming they belong to *D. magna* species (File S1). Genetically homogeneous *D. magna* were obtained from a single mother. The sequential clones were kept in Rocchetta© water at 20 ± 1 °C with a photoperiod of 16 h light (100 lx): 8 h dark for at least two months until testing. They were fed three times a week with *Scenedesmus dimorphus* (8 × 10^5^ cells mL^−1^). *S. dimorphus* culture method details have been previously reported [91]. The well-being of the population was regularly evidenced by the low mortality (≤2% per week), the high reproduction rate (approximately 15 newborns per clutch), and the absence of winter eggs (ephippia) and/or males. *D. magna* clone’s sensitivity was checked every four months by exposure to potassium dichromate [92].

### 4.3. Daphnia magna Reproduction Test

In order to have the number of organisms necessary to conduct the biomolecular investigations, some modifications to the OECD 211 protocol [93] were necessary. Instead of using 10 daphnia individually held in 10 beakers for each exposure level and control, 100 daphnia were used, divided into two groups of 50, each placed in a vessel containing 750 mL of medium. This holding condition, which we normally use for culturing *D. magna*, has long been shown to have no undesirable effects on its health condition (see previous section). The crustaceans were exposed to two predetermined concentrations of FLU, i.e., 2.0 mg L^−1^, given as the effect concentration and at a tenfold dilution (0.2 mg L^−1^); one group was maintained in pure medium, serving as a negative control. The higher concentration was selected based on results from a previous study [31] where only moderate, non-significant phenotypic effects were observed at this exposure level (20% lethality and 20.6 reproduction inhibition). The lower concentration was adopted with the aim of evidencing any possible biomolecular alteration despite the predictable absence of phenotypic effects. The culture medium of each group of 50 + 50 daphnids was renewed three times a week, recording each time the number of dead and newborn individuals, which were collected and removed. At the end of the test (i.e., 21 days), a portion of the daphnids were subjected to absolute ethanol fixation to measure body growth. The remaining organisms were harvested, their brood pouch was emptied (to avoid extracting RNA originating from eggs), then they were flash-frozen in liquid nitrogen for subsequent RNA extraction.

### 4.4. RNA Isolation and RNA Sequencing

To understand the molecular effects that FLU might have on the transcriptome of *D. magna*, 21-day-old daphnids were grouped into 6 organisms per pool, obtaining 3 pools for each experimental group. Total RNA was isolated with the Direct-zol^TM^ RNA Microprep kit (ZYMO RESEARCH, Irvine, CA, USA), following the protocol provided by the manufacturer. The purity and quantity of RNA were first measured with a NanoDrop ND1000 spectrophotometer (Thermo Fisher Scientific, Waltham, MA, USA) and then confirmed by fluorometric quantification with Qubit^TM^ 4 Fluorometer (ThermoFisher Scientific, Singapore). Total RNA integrity was assessed on a 4150 TapeStation System (Agilent Technologies, Santa Clara, CA, USA). RNA samples were shipped on dry ice to BGI (BGI Genomics, Company Limited Hong Kong, Hong Kong). RNA-seq libraries were constructed following a polyA selection approach and using the BGI strand-specific transcriptome library construction protocol (DNBSEQ). Sequencing was then conducted through the DNBSEQ PE100 platform at BGI Genomics. The output was about 270 million good-quality 100 bp paired-end reads. Raw data were deposited in GeneBank under the BioProject PRJNA939942.

### 4.5. Transcriptome Mapping and Differential Expression Analysis

Initial quality control was performed using FastQC software version 0.11.9 [94]. Prior to mapping the reads onto the *D. magna* genome, raw reads were filtered and cleaned by using Trimmomatic 0.36. [95] to get rid of adapters and low-quality sequences. After trimming, the success of the mRNA selection step performed during libraries’ preparation was confirmed by evaluating the percentages of ribosomal RNA (rRNA) reads, using the SortMeRNA version 4.3.4 tool [96]. These percentages were negligible in all the sequenced libraries. Then, surviving reads were mapped twice against *D. magna* genome, first using the genome provided by EnsemblMetazoa (GCA_001632505.1; https://metazoa.ensembl.org; accessed on 7 February 2022;), and the second time using the genome available on NCBI platform (GCA_020631705.2; https://www.ncbi.nlm.nih.gov; accessed on 18 March 2022). Since the NCBI genome was found to be more accurately annotated, gene counts obtained using this genome were selected for the following analysis. The STAR aligner [97] version 2.5.3a was used for the mapping process, following the two-pass mode and setting the maximum allowed number of multiple alignments and the maximum number of mismatches to 10 and 8, respectively. Read counts for each sample, at the gene level, were extracted by setting the *GeneCounts* quantification while running STAR.

The mapping output underwent differential expression analysis in R studio (R version 4.1.1) [98]. To test whether exposure of *D. magna* to FLU could induce transcriptional changes, pairwise comparisons were performed between daphnids exposed to flumequine 0.2 or 2.0 mg L^−1^, hereinafter referred as FLU-L and FLU-H, and daphnids reared in pure medium (CTRL). The differentially expressed genes (DEGs) were determined by using the Likelihood Ratio test (LRTest) implemented in EdgeR [99], setting the following contrasts: FLU-L vs. CTRL, FLU-H vs. CTRL. A minimum false discovery rate (FDR) of 0.05 and a minimum log fold-change (LFC) of 0.6 (i.e., FC of 1.5) were used as thresholds of significance between exposed and control daphnids.

### 4.6. Enrichment Analysis

The GO annotations needed for the functional analysis were obtained as follows: the Entrez feature Batch Entrez (provided by the National Center for Biotechnology Information NCBI) allowed us to retrieve the protein sequences by importing the list of NCBI gene IDs. Then, by means of a free annotation web server (pannzer2), we retrieved Gene Ontology (GO) associated to each protein [100].

In order to assess whether exposure to FLU could significantly affect specific gene pathways, all DEGs found in each pairwise comparison were analyzed with R package ClusterProfiler version 4.2.1 [101], specifically using the GO over-representation test *enrichGO*. Finally, a gene set enrichment analysis (GSEA) was performed using the tool provided by the ClusterProfiler package. For this computational analysis, the input data were manually prepared starting from the lists of genes produced by the LRTest function. Specifically, all genes were preranked according to their *p*-value using “1-Pvalue” and “-(1-*p*-value)” to include the direction of their expression in the analysis (up- or downregulation, respectively).

### 4.7. Quantitative Real-Time PCR (qPCR)

Five target transcripts among the top up- and downregulated genes (i.e., cuticle protein 18.6, larval cuticle protein 2-like, larval cuticle protein F1, vitelline membrane protein Vm26Ab, vitellogenin 2) and two internal control genes (ICGs, actin and glyceraldehyde-3-phosphate dehydrogenase 2) were chosen for qPCR validation of RNA-seq data. For each target transcript, gene-specific primers (Table S5) were designed using the Primer3 (version 4.1.0) web service (https://primer3.ut.ee/; accessed on 26 April 2023). Primer specificity was evaluated either in silico by means of the BLAST tool (https://blast.ncbi.nlm.nih.gov/Blast.cgi; accessed on 26 April 2023) or experimentally by melting curve analysis [102].

The same RNA used for the RNA-seq experiments was used for confirmatory qPCR analyses. First-strand cDNA was synthesized from 1.5 µg of total RNA using the High-Capacity cDNA Reverse Transcription Kit (Life Technologies, Carlsbad, CA, USA). qPCR reactions (10 µL final volume) consisted of 1X Power SYBR Green PCR Master Mix (Life Technologies, Carlsbad, CA, USA), 150 or 300 nM forward and reverse primers (Table S6), and 0.75 ng cDNA. qPCR analysis was performed in duplicate in a Stratagene MX3000P thermal cycler (Agilent Technologies, Santa Clara, CA, USA) using standard PCR conditions. The quality of each qPCR assay (Table S6) was gathered from standard curve slopes and linear correlation coefficients. The PCR efficiency (E%) was measured by the equation E = ((10^−1/slope^)−1) ×100 and was considered acceptable with values between 90% and 110%. ICG assay parameters were comparable to that of the target genes (Table S6); moreover, no statistically significant differences were observed in their expression profile between CTRL and treated samples. Messenger RNA relative quantification was performed using the ∆∆Ct method [103], using the arithmetic mean of the two selected ICGs and a cDNA pool comprehending external samples as calibrator. Data are expressed as fold-change of treated (FLU-L and FLU-H) versus CTRL samples ± mean standard error (SEM).

### 4.8. Data Analysis

One-way ANOVA was used to infer differences in survival, growth, and reproduction between the control group and the FLU-exposed groups in the chronic test of each generation. A Tukey’s multi-comparison test was then used to compare the group means. Values of *p* ≤ 0.05 were considered significant.

## 5. Conclusions

Despite the high public and scientific interest in a suitable environmental risk framework for FQs, the molecular effects of these antibiotics are still scarcely known. To the best of our knowledge, this is the first scientific report on the transcriptional effects of FLU in the non-target aquatic species *D. magna*. Under the umbrella of ecotoxicology, we coupled phenotypic and omics approaches showing that FLU, at the selected concentrations, might have a significant impact on Daphnia’s survival, growth, reproduction, and transcriptome. More importantly, although a detailed annotation of the *D. magna* genome is still lacking, the omics approach we adopted in this study proved to be an appropriate tool for detecting subtle toxic effects that may not be apparent at the phenotypic level.

Subsequent studies may further clarify whether the molecular effects reported in the first generation may also be observed in the following generations, possibly involving epigenetic changes that could be inherited through generations.

## Figures and Tables

**Table 1:** Phenotypical results of the OECD 211 test. Data are expressed as mean of two independent batches of daphnids, each consisting of 50 organisms. *: *p* ≤ 0.05 (one-way ANOVA followed by Tukey’s test). FLU-L: 0.2 mg L^−1^; FLU-H: 2.0 mg L^−1^.

Group	Mortality Rate (%) N = 100	Daily Newborns per Parent Animal at the Start of the Test	Reproduction Inhibition Rate (%)	Daily Growth (Length, µm)	Daily Growth Inhibition (%)
CTRL	6	3.62 ± 1.86		130.41 ± 12.98	
FLU-L	9	3.45 ± 1.73	4.56	129.12 ± 6.52	1.00
FLU-H	46 *	1.94 ± 1.79	46.50 *	118.89 ± 8.43	8.82 *

## Data Availability

The raw sequencing data have been deposited in GenBank under the BioProject’s accession number PRJNA939942.

## Supplementary Materials

The following supporting information can be downloaded at: https://www.mdpi.com/article/10.3390/ijms24119396/s1, References [104,105,106,107] are cited in the supplementary materials.
