# Supplementary material for: An In Vivo Whole-Transcriptomic Approach to Assess Developmental and Reproductive Impairments Caused by Flumequine in Daphnia magna
Source: Int J Mol Sci. 2023 May 28;24(11):9396. doi: 10.3390/ijms24119396 (PMC10253896; doi:10.3390/ijms24119396)
Supplement: Supplementary file 1 [file ijms-24-09396-s001.zip › FigureS1_rev.pdf]

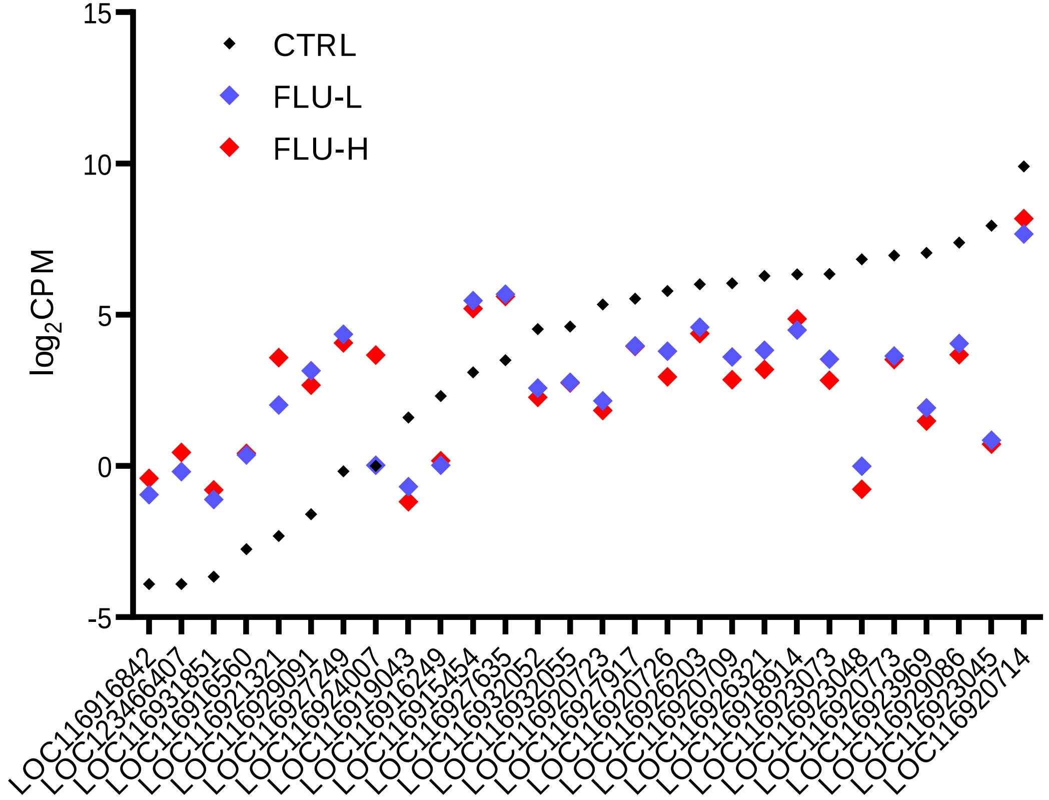

Figure S1. Expression levels of common DEGs (here identified by the locus name) in the two experimental groups compared to controls. Data are expressed as normalized $\log_2$CPM (Count Per Million). FLU-L: 0.2 mg L-1; FLU-H: 2.0 mg L-1.
