# Supplementary material for: An In Vivo Whole-Transcriptomic Approach to Assess Developmental and Reproductive Impairments Caused by Flumequine in Daphnia magna
Source: Int J Mol Sci. 2023 May 28;24(11):9396. doi: 10.3390/ijms24119396 (PMC10253896; doi:10.3390/ijms24119396)
Supplement: Supplementary file 1 [file ijms-24-09396-s001.zip › FigureS2_rev.pdf]

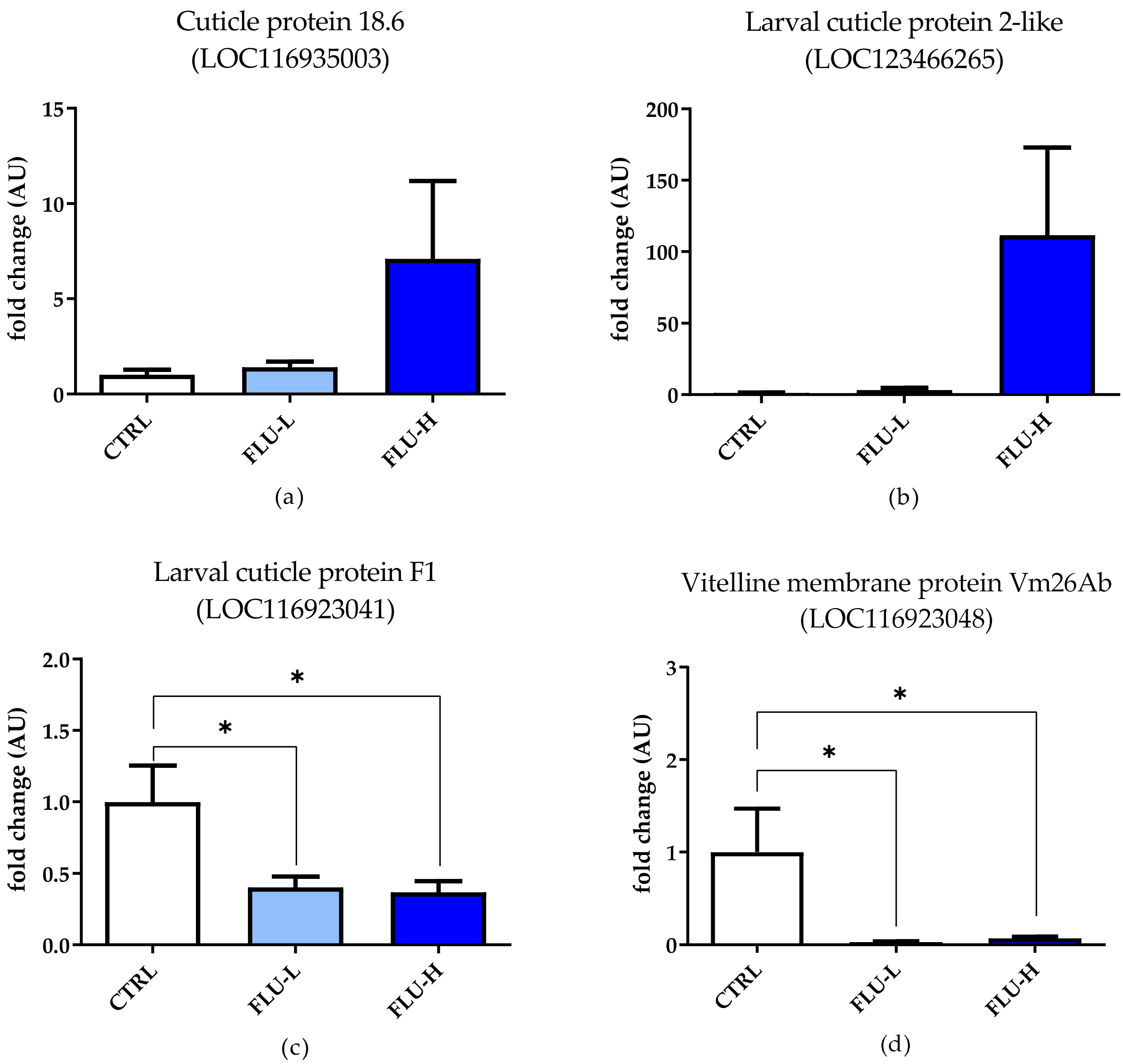

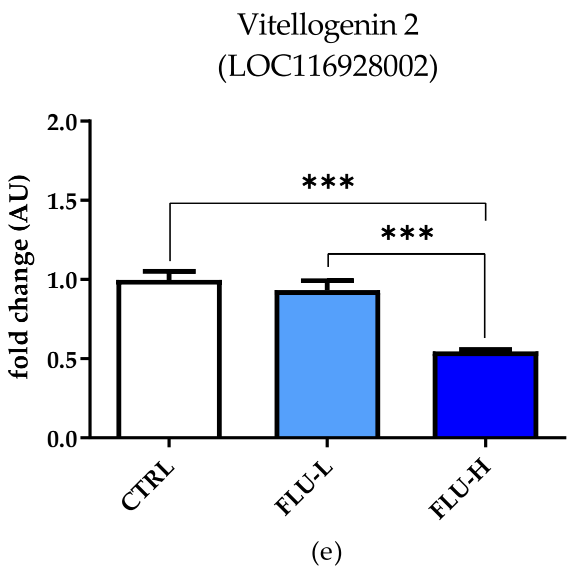

	RNA-seq (log_2FC)		qPCR (logFC)	
	FLU-L	*FLU-H*	*FLU-L*	*FLU-H*
**LOC116935003**	6.35	11.81	1.40	7.09
**LOC123466265**	2.3	6.83	3.05	111.62
**LOC116923041**	-1.29	-2.43	-2.50	-2.71
**LOC116923048**	-6.83	-7.59	-37.95	-14.68
**LOC116928002**	0.01	-1.84	-1.07	-1.83

(f)

Figure S2. qPCR validation of RNA-seq data. The mRNA expression of five target genes (a-e) was assessed by qPCR. Data are expressed as fold-change (arbitrary units, AU) versus CTRL, and given as mean ± standard error (SEM) of three or four samples (control and treated groups, respectively). *: P ≤0.05, ***: P ≤0.001 (one-way ANOVA followed by Tukey's test). FLU-L: 0.2 mg L-1; FLU-H: 2.0 mg L-1. (f) Comparison of RNA-seq and qPCR results in terms of logaritmich Fold Changes. Statistically significant results are reported in green (significant up-regulation) or red (significant down-regulation) colour.
