# Supplementary material for: An In Vivo Whole-Transcriptomic Approach to Assess Developmental and Reproductive Impairments Caused by Flumequine in Daphnia magna
Source: Int J Mol Sci. 2023 May 28;24(11):9396. doi: 10.3390/ijms24119396 (PMC10253896; doi:10.3390/ijms24119396)
Supplement: Supplementary file 1 [file ijms-24-09396-s001.zip › FileS1_rev.pdf]

Supplementary file S1

Daphnia magna species identification

The purified DNA was isolated from a pool of 3 daphnids homogenized with mortar and pestle in ice. DNA was isolated using the DNeasy kit Blood and Tissue (Qiagen) according to the manufacturer's instructions. Then, DNA was quantified by the NanoDrop spectrophotometer 1000D v. 3.8.1 (ThermoFisher Scientific, US), resulting in 580 ng/μ. A new pair of primers was designed to specifically amplify and sequencing *D. magna* mitochondrial gene Cytochrome C Oxidase subunit I (COI). Primers were designed using the free software Primer 3plus [102].

Notably, two sets of primers [104,105] targeting COI in Metazoan and Zooplancton organisms were preliminary assessed but their alignment against *D. magna* mitochondrial reference sequence (NC_026914.1) produced a large number of mismatches. For this reason, we designed a couple of specific primers. Primers sequences are reported below:

Forward primer: CAACCAATCATAAGGACATTGG
Reverse primer: TACACTTCAGGGTGGGGCCAAAGAATC

Around 20 ng of DNA was amplified in a thermalcycler Proflex (Applied Biosystems™ Proflex PCR System) by using the Phire Hot Start II DNA Polymerase (ThermoFisher Scientific, US) and the following reagents: 5 μl of 5X buffer, 0.5 μl of dNTPs 10 nM, 1.25 μl for each 2 primer 10um, 0.5 μl of the polymerase 50X, DNA, and water q.b. to 25 μl. The thermal protocol used was as follows: 98°C x 30sec; 38 cycles of: 98°C x 8 sec; 59°C x 8sec; 72°C x 8sec; 72°C x 2min. The amplicons were subjected to an agarose gel electrophoresis (1.5% agarose in TBE buffer) in an Electrophoresis System RunOne device (Embi Tec, US), at 100 volts for 20 minutes; a SHARPMASS 100 (EuroClone S.p.A., Italy) DNA ladder was used; then the gel was visualized on a GelDoc System (Bio-rad Laboratories, US). The amplicon length was close to the 700 bp ladder band (expected amplicon length: 706 bp).

A volume of 50 ul of the obtained amplicons has been purified by a silica column in kit (NucleoSpin Gel and PCR Clean up, Mini kit for gel extraction and cleaning PCR, Macherey-Nagel), according to the producer's instructions; each sample has been eluted in 20 μl of the elution buffer. The purified samples (1.5 μl in a final volume of 8 μl) was processed with BrilliantDye™ Terminator (v1.1) Cycle Sequencing Kit (NimaGen, CG Nijmegen, NL) on an AB3730xl DNA Analyzer (Applied Biosystems™, ThermoFisher Scientific, US), at the BMR Genomics Sequencing Core (spin-off of Padua University). The .abi sequences (forward and reverse strand). The obtained sequence is reported below:

ATAAGGACATTGGGACTTTATATTTTGTATTCGGGGTCTGATCAGGTATGGTAGGAACTGCTTTAAGTATACTTATTCGAGCTGAGT
TAGGGCAATCAGGTAGTTTAATTGGGGATGATCAGATTTATAATGTTATTGTCACTGCCCACGCGTTTGTAATAATTTTTTTTATGG
TTATGCCTATTATAATTGGAGGTTTCGGGAACTGGCTAGTACCTTTGATGTTGGGAGCTCCTGACATAGCCTTCCCTCGATTAAATA
ATTTAAGTTTTTGATTTTTACCCCCTGCTTTAACTCTTTTACTTGTGGGAGGGGCAGTGGAAAGTGGTGCTGGGACTGGTTGAACTG
TATACCCCCCTCTCTCTGCGGGGATCGCTCATGCTGGGGCCTCTGTTGACTTAAGTATCTTTTCTCTGCATCTAGCAGGTGTTTCTTC
TATTTTAGGGGCAGTAAATTTTATTACAACTATCATTAATATACGATCTTTAGGTATAACTTTAGATCGAATTCCCTTGTTTGTATGA
GCGGTTGGAATCACTGCACTCTTACTTTTACTAAGTTTGCCCGTTCTTGCGGGAGCAATTACCATACTCTTAACTGACCGTAATTTGA
ATACTTCATTCTTTGATCCTGCGGGGGGTGGGGATCCAATTTTATACCAACATTTATTTTGATTCTTTGGCCACCC

The obtained COI sequence was aligned by BOLDSYSTEM [106]. BOLD is an archiving and analysis platform through which animal identification (COI) is possible. As stated on the site, sequences from the 5' region of the mitochondrial COI gene are accepted, and when possible, BOLD ID returns a species-level identification. Based on the BOLD alignment we confirm that the daphnids used in the present study belong to the *D. magna* species with a probability of placement of 100%.
