# Supplementary material for: An In Vivo Whole-Transcriptomic Approach to Assess Developmental and Reproductive Impairments Caused by Flumequine in Daphnia magna
Source: Int J Mol Sci. 2023 May 28;24(11):9396. doi: 10.3390/ijms24119396 (PMC10253896; doi:10.3390/ijms24119396)
Supplement: Supplementary file 1 [file ijms-24-09396-s001.zip › TableS5_rev.pdf]

Table S5. List of genes selected for qPCR validation of RNA-seq data: Gene Locus and description, NCBI sequence ID, primer sequences, amplicon size and reference.

Gene Locus	Gene description	NCBI sequence ID	Primer sequence (5'-3')	Amplicon size (bp)	Reference
LOC116935003	Cuticle protein 18.6	XM_032942392	F: GCCCATTCCGAGAGCAGTAA<br>R: ACGATGGTGTTGGCCTGTAT	169	Designed ex novo
LOC123466265	Larval cuticle protein 2-like	XM_032920616	F: TCTGACTACACTACCCGCGA<br>R: TCCAGCATCATCAGCAACCC	180	Designed ex novo
LOC116923041	Larval cuticle protein F1	XM_032929579	F: CCTGATGATCTTGGCTGTTTTGG<br>R: CAGCGGCGTAGGGAAGAC	101	Designed ex novo
LOC116923048	Vitelline membrane protein Vm26Ab	XM_032929586	F: TGCCTCCATCTCCTTATTCCG<br>R: CGTATGGATCGGAAGCAGCC	138	Designed ex novo
LOC116928002	Vitellogenin 2	XM_045180640	F: GACCTCCCCACTTGCTACAC<br>R: TTCAGTTCGAGAGTGTGGCC	140	Designed ex novo
LOC116919128	Actin, muscle	XM_032925012	F: GCCCTCTTCCAGCCCTCATTCT<br>R: TGGGGCAAGGGCGGTGATTT	189	Wei et al. (2022)*
LOC116919264	Glyceraldehyde-3-phosphate dehydrogenase 2	XM_032925227	F: TGCTGATGCCCCAATGTTTGTTGT<br>R: GCAGTTATGGCGTGGACGGTTGT	174	Wei et al. (2022)*

* [107]
