# Supplementary material for: An In Vivo Whole-Transcriptomic Approach to Assess Developmental and Reproductive Impairments Caused by Flumequine in Daphnia magna
Source: Int J Mol Sci. 2023 May 28;24(11):9396. doi: 10.3390/ijms24119396 (PMC10253896; doi:10.3390/ijms24119396)
Supplement: Supplementary file 1 [file ijms-24-09396-s001.zip › TableS6_rev.pdf]

Table S6. qPCR assay parameters: primer concentration, efficiency, linear regression coefficient (R squared) and dynamic range.

Gene	Primer concentration	Efficiency (%)	Linear regression coefficient (R^2)	Dynamic range (Ct)
Cuticle protein 18.6 (LOC116935003)	150F/150R	95.9	0.996	25.90 – 33.87
Larval cuticle protein 2-like (LOC123466265)	150F/150R	96.4	1.000	20.84 – 32.23
Larval cuticle protein F1 (LOC116923041)	300F/300R	99.4	1.000	16.90 – 28.07
Vitelline membrane protein Vm26Ab (LOC116923048)	300F/300R	99.0	0.999	21.86 – 32.90
Vitellogenin 2 (LOC116928002)	150F/150R	101.7	0.998	14.66 – 24.00
Actin, muscle (LOC116919128)	300F/300R	100.0	0.999	16.94 – 27.91
Glyceraldehyde-3-phosphate dehydrogenase 2 (LOC116919264)	300F/300R	99.5	0.999	16.42 – 27.71

F: forward primer; R: reverse primer.
